# Plasma glutamate carboxypeptidase is a negative regulator in liver cancer metastasis

**DOI:** 10.18632/oncotarget.12967

**Published:** 2016-10-28

**Authors:** Jae-Hye Lee, Hyun-Soo Cho, Jeong-Ju Lee, Soo Young Jun, Jun-Ho Ahn, Ju-Sik Min, Ji-Yong Yoon, Min-Hyuk Choi, Su-Jin Jeon, Jung Hwa Lim, Cho-Rok Jung, Dae-Soo Kim, Hyun-Taek Kim, Valentina M. Factor, Yun-Han Lee, Snorri S. Thorgeirsson, Cheol-Hee Kim, Nam-Soon Kim

**Affiliations:** ^1^ Genome Research Center, Korea Research Institute of Bioscience and Biotechnology, Daejeon 305-333, Republic of Korea; ^2^ Gene Therapy Research Unit, Korea Research Institute of Bioscience and Biotechnology, Daejeon 305-333, Republic of Korea; ^3^ Department of Functional Genomics, Korea University of Science and Technology, Daejeon 305-333, Republic of Korea; ^4^ Department of Biology, Chungnam National University, Daejeon 305-764, Republic of Korea; ^5^ Laboratory of Molecular Pharmacology, Center for Cancer Research, NCI, NIH, Bethesda, MD 20892-5068, USA; ^6^ Department of Molecular Medicine, Keimyung University School of Medicine, Daegu 704-701, Republic of Korea; ^7^ Laboratory of Human Carcinogenesis, Center for Cancer Research, NCI, NIH, Bethesda, MD 20892-4255, USA

**Keywords:** liver cancer, metastasis, PGCP, Wnt/β-catenin

## Abstract

Tumor metastasis is the leading cause of cancer death. In the metastatic process, EMT is a unique phenotypic change that plays an important role in cell invasion and changes in cell morphology. Despite the clinical significance, the mechanism underlying tumor metastasis is still poorly understood. Here we report a novel mechanism by which secreted plasma glutamate carboxypeptidase(PGCP) negatively involves Wnt/β-catenin signaling by DKK4 regulation in liver cancer metastasis. Pathway analysis of the RNA sequencing data showed that PGCP knockdown in liver cancer cell lines enriched the functions of cell migration, motility and mesenchymal cell differentiation. Depletion of PGCP promoted cell migration and invasion via activation of Wnt/β-catenin signaling pathway components such as phospho-LRP6 and β-catenin. Also, addition of DKK4 antagonized the Wnt/β-catenin signaling cascade in a thyroxine (T4)-dependent manner. In an *in vivo* study, metastatic nodules were observed in the lungs of the mice after injection of shPGCP stable cell lines. Our findings suggest that PGCP negatively associates with Wnt/β-catenin signaling during metastasis. Targeting this regulation may represent a novel and effective therapeutic option for liver cancer by preventing metastatic activity of primary tumor cells.

## INTRODUCTION

Liver cancer ranks as the third leading cause of cancer death worldwide [1]. Tumor metastasis causes 90% of the deaths attributable to cancer. The processes involved in liver cancer progression are very complex, and several deregulated signaling pathways participate in tumorigenesis and metastasis of liver cancer. Despite its immense clinical significance, molecular mechanisms underlying the metastasis and metastasis-inducing factors remain to be examined [2].

Epithelial-mesenchymal transition (EMT) is a unique phenotypic change that occurs during the process of tumor metastasis. EMT is characterized by a loss of epithelial features, including the reduction of E-cadherin, cytokeratin and claudin, while gaining the properties of mesenchymal cells, such as upregulation of N-cadherin, fibronectin and vimentin. EMT has been shown to contribute to cell invasion and therapeutic resistance through regulation of several transcription factors (Twist, Snail and ZEB), which are commonly induced in invasive tumors [2–6]. Liver cancers recurrently exhibit proximal metastasis leading to intracerebral and intrahepatic colonization [7]. Reduction of E-cadherin expression at cell junction and cytoplasmic localization of E-cadherin are frequently found in a metastatic patient group with poorly differentiated liver cancer. In contrast, Twist, a negative regulator of E-cadherin, is abundantly expressed in metastasis and leads to tumor invasion [7]. Blocking the function of transforming growth factor-β (TGF-β) with LY2109761 inhibitor up-regulated E-cadherin and reduced migration and invasion of liver cancer cells [8].

Various signaling pathways regulate the expression of the metastasis-related genes in liver cancer. Among these is Wnt/β-catenin signaling pathway, shown to promote cell migration and invasion in several types of cancer [9–11]. A key role in Wnt/β-catenin activation plays Wnt co-receptor LRP5/6. Along with Frizzled, phosphorylated LRP5/6 binds to Wnt thus triggering Wnt/β-catenin signaling cascade in cytoplasm [12]. Subsequently, active β-catenin (unphosphorylated by GSK3β and not degraded in a complex comprising APC/Axin/GSK3β/β-catenin) translocates to the nucleus and induces transcriptional activation of metastasis-related genes leading to the acquisition of mesenchymal phenotype. In liver cancer xenograft model, it was demonstrated that depletion of β-catenin reduced the abdominal metastatic lesions [13]. On the other hand, Dickkopf (DKK) family is a known antagonist of the Wnt/β-catenin signaling blocking tumor cell migration and invasion [14–17].

Plasma glutamate carboxypeptidase (PGCP) is a secreted protein hydrolyzing the circulating peptides in the extracellular microenvironment. In particular, PGCP liberates I-thyroxine (T4) from thyroglobulin (Tg), converted to 3,5,3′-triiodothyronine (T3) by type I 5′-deiodinase (DIO1) [18, 19]. The thyroid hormone T3 binds to thyroid hormone receptors (TRs), and after heterodimerization with retinoid X receptor (RXR) can act as a transcription factor mediating diverse physiological processes, such as embryonic development, cell differentiation, metabolism and cell proliferation, by regulating expression of the downstream target genes. Notably, T3 induced the expression of DKK4, a well-known antagonist of Wnt/β-catenin signaling pathway, and suppressed cell invasion in liver cancer cells suggesting a role of T3 as a tumor suppressor [14, 15]. PGCP is one of the secreted or plasma proteins upregulated in more than half of the hepatitis C virus (HCV)-associated HCCs (hepatocellular carcinoma) as compared with the adjacent non-tumoral cirrhotic tissues [20]. However, the roles of PGCP contributing to proliferation and metastasis are not yet fully defined.

Given this information, we hypothesized that PGCP may be a negative regulator of liver cancer metastasis by inducing DKK4 expression and thereby blocking Wnt/β-catenin signaling. Our results provide *in vitro* and *in vivo* evidence that PGCP depletion promoted cell migration and invasion via activation of Wnt/β-catenin signaling cascade. DKK4 antagonized the effect in a T4-dependent manner. In addition, the expression pattern of PGCP in liver cancer tissues was inversely correlated with β-catenin expression. Thus, our findings provide a novel mechanism of PGCP-mediated negative regulation of liver cancer metastasis potentially representing an effective target for cancer therapy.

## RESULTS

### Inhibition of PGCP expression promotes migration and invasion of liver cancer cells

**Figure 1:**
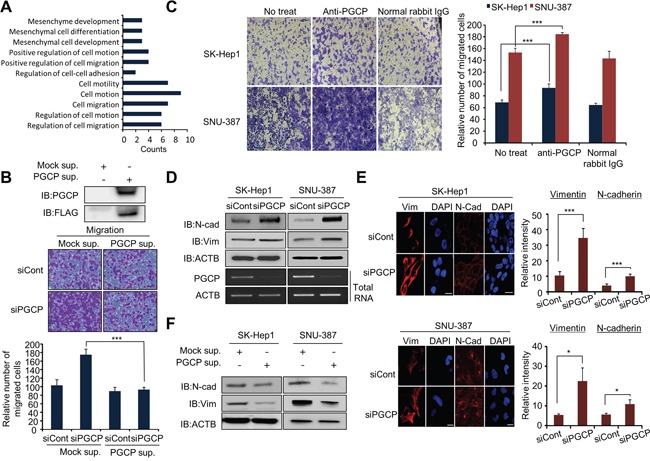
PGCP silencing induces migration and invasion in SK-Hep1 and SNU-387 cells **A.** Metastasis-related genes associated with PGCP knockdown were analyzed using the DAVID gene ontology program. The enriched terms are shown. **B.** Migration assay with PGCP supernatant. Top, Western blot analysis after transfection with FLAG-Mock or FLAG-PGCP, respectively. After 24 h, the PGCP protein secreted to culture media was concentrated using an Amicon column. The samples were immunoblotted with anti-FLAG and anti-PGCP antibodies. Bottom, SK-Hep1 cells were treated with siRNAs (siCont and siPGCP) for 48 h, and secreted PGCP was added to the culture media. Cell migration assays was performed after24 h. The *p* value was calculated using Student's *t*-test (***p<0.001). **C.** Migration assay with anti-PGCP antibody. After siRNA treatment, anti-PGCP antibody (2 ng/ml; SK-Hep1, 1 ng/ml; SNU-387) was added to culture media for 24 h. Cells were loaded into transwell inserts and invasion chambers, respectively. Migrated and invasive cells were fixed with methanol and stained with crystal violet (Left). The *p* value was calculated using Student's *t*-test (***p<0.001) (Right). **D.** Western blot analysis of vimentin (Vim) and N-cadherin (N-cad) after treatment with siCont or siPGCP for 48 h. Anti-actin (ACTB) antibody was used as an internal control. **E.** Immunofluorescence of Vimentin and N-cadherin. SK-Hep1 and SNU-387 cells treated with siCont or siPGCP were fixed with methanol, and stained with anti-vim and anti-N-cad (Alexa Fluor 594, red) antibodies and DAPI (blue). Scale bar, 20 μm (Left). The signal intensities corresponding to Vimentin and N-cadherin were quantified using the ImageJ software (Right). The *p* value was calculated using Student's *t*-test (*p<0.05, ***p<0.001). **F.** Western blot analysis of Vimentin and N-cadherin after treatment with PGCP supernatant. Anti-ACTB antibody was used as an internal control.

To address the biological functions of PGCP in liver cancer progression, SK-Hep1 cells were transfected with PGCP siRNA (siPGCP), and then RNA sequencing was employed to compare the global gene expression profiles of PGCP-deficient cells versus cells transfected with a negative control siRNA (siCont). Pathway analysis of the molecular signature revealed that PGCP knockdown enriched the functions involved in mesenchymal cell differentiation, cell motility, and cell migration (Figure 1A). Accordingly, PGCP knockdown also caused a down-regulation of the epithelial cell markers *CDH1, DSP, OCLN* and *CLDN1*, and an up-regulation of mesenchymal cell markers *CDH2, FN1, COL1A1, SNAI2* and *FOXC2* (data not shown).

siRNA silencing of PGCP significantly increased the rate of cell migration and invasion in both examined tumor cell lines (SK-Hep1 and SNU-387) as compared to the cells treated with siCont (Supplementary Figure S1). As PGCP is a secretory protein hydrolyzing circulatory peptides in serum [18, 21], we then collected the supernatant from the FLAG-tagged PGCP overexpressed 293T cells and assessed its effect on cell migration. As shown in Figure 1B, supernatant collected from PGCP-overexpressed cells significantly decreased the migration ability of SK-Hep1 cells by PGCP knockdown. The siPGCP-mediated increase in cell migration and invasion was reversed by the PGCP supplementation in a dose-dependent manner (Supplementary Figure S2). Conversely, treatment with anti-PGCP antibody enhanced the migration ability of both SK-Hep1 and SNU-387 cells when compared with cells treated with normal rabbit IgG (Figure 1C).

Since cancer cells gain mesenchymal characteristics to initiate metastasis [3], we next examined whether depletion of PGCP could influence the expression of EMT and mesenchymal-epithelial transition (MET) markers. siRNA knockdown of PGCP moderately increased expression of E-cadherin, vimentin and N-cadherin in SK-Hep1 and SNU-387 cells on the mRNA (Supplementary Figure S3) and protein levels (Figure 1D and Figure 1E), whereas PGCP supplementation repressed this effect (Figure 1F). Taken together, these data strongly indicate that secreted PGCP protein suppresses migration in liver cancer cells by regulating the EMT marker expression.

### Depletion of PGCP stabilizes β-catenin via inactivation of GSK3β in metastasis

**Figure 2:**
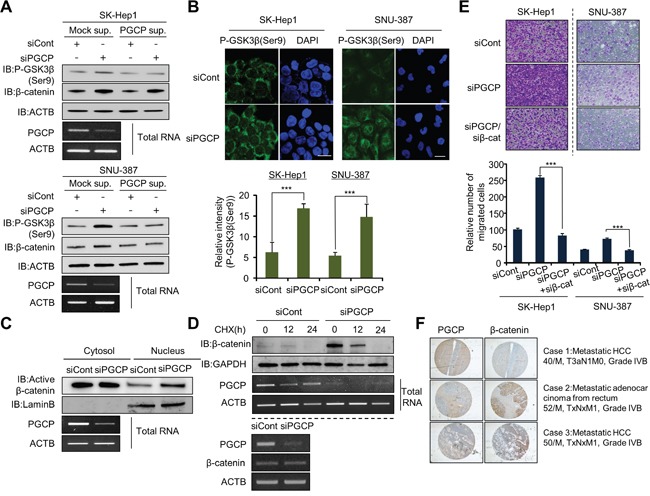
PGCP knockdown increases phosphorylation of GSK3β **A.** Western blot analysis of phospho-GSK3β (Ser9) and β-catenin. PGCP supernatant was added to cultured SK-Hep1 and SNU-387 cells treated with siCont or siPGCP. Whole cell lysates were prepared and analyzed by western blotting using the indicated antibodies. ACTB was used as a loading control. **B.** Immunofluorescence of phospho-GSK3β (Ser9). The cells treated with siCont or siPGCP were fixed with methanol and stained with anti-phospho-GSK3β (Ser9) (Alexa Fluor 488, green) antibody and DAPI (blue). Scale bar, 20 μm. The signal intensities corresponding to phospho-GSK3β (Ser9) were quantified using the ImageJ software (bottom). The p values were calculated using Student's t-test (***p<0.001. **C.** Western blot analysis of β-catenin. The whole cell lysates were prepared form SK-Hep1 cells treated with siCont or siPGCP and separated into nuclear or cytosolic fractions using a nuclear and cytoplasmic extraction reagent (NE-PER, Thermo). The purity of cell fractionation was monitored by Lamin B. **D.** Detection of PGCP and β-catenin by Western blotting and RT-PCR. Top, Cells were treated with 100 μg/ml of cycloheximide (CHX) for the indicated times and immunoblotted to examine β-catenin levels after treatment with siCont or siPGCP. Anti-GAPDH antibody was used as an internal control. Bottom, The expression level of PGCP and β-catenin was measured by semi-quantitative RT-PCR after PGCP depletion in SK-Hep1 cells. ACTB was used as an internal control. **E.** Cell migration assays in SK-Hep1 and SNU-387 cells. After 24 h, migrated cells were fixed with methanol and stained with crystal violet. The p value was calculated using Student's t-test (***p<0.001). **F.** Inverse correlation between expression of PGCP and β-catenin in human liver cancer tissues. Immunohistochemical analysis of PGCP and β-catenin. Liver cancer samples were purchased from SUPER BIO CHIPS. Negative correlation of staining between PGCP and β-catenin was calculated using Spearman's correlation coefficient by ranks with ties.

During tumor metastasis, several signaling pathways (e.g., AKT, Wnt and NF-kB) contribute to EMT and cell invasion by activating transcription factors SNAIL, SLUG and ZEB1 [22, 23]. To define a role for PGCP in metastasis, we examined the phosphorylation status of key signaling molecules in each pathway after PGCP inactivation in liver cancer cells. PGCP knockdown in SK-Hep1 cells did not influence the phosphorylation status of AKT (Supplementary Figure S4A) as well as IkBα phosphorylation even after TNF-α stimulation (Supplementary Figure S4B). Instead, loss of PGCP function significantly increased GSK3β (Ser9) phosphorylation and β-catenin expression of both in SK-Hep1 and SNU-387 cells (Supplementary Figure S4C). The addition of overexpressed PGCP to the culture media of SK-Hep1 reversed the effects induced by PGCP silencing (Figure 2A). Immunofluorescence staining confirmed that PGCP knockdown increased the phosphorylation of GSK3β (Ser9) in SK-Hep1 and SNU-387 cells (Figure 2B). We further found that most of the active β-catenin was translocated from cytosol to nucleus in the PGCP knockdown cells as compared with cells treated with siCont (Figure 2C), indicating that PGCP depletion stabilized β-catenin. And experiments with cycloheximide (CHX), an inhibitor of protein biosynthesis, showed that si-mediated inactivation of PGCP in SK-Hep1 is slowed down β-catenin degradation (Figure 2D, top). However, the β-catenin mRNA levels were comparable in siCont- and siPGCP-treated cells (Figure 2D, bottom). To confirm the functional correlation of PGCP with β-catenin, we performed a cell migration assay after co-transfection with siPGCP and siβ-catenin (siβ-cat). The PGCP knockdown-mediated increase in cell migration was significantly abolished by the complimentary siβ-cat silencing (Figure 2E). Finally, we examined the pattern of PGCP and β-catenin expression in 42 human liver cancer tissues using immunohistochemistry. We found a reverse correlation (r= −0.33336, *P*=0.02) between the PGCP and β-catenin expression as shown by the depletion of PGCP based on Spearman's rank correlation coefficient analysis (Figure 2F). These results suggest that PGCP depletion stabilizes β-catenin via inactivation of GSK3β and promotes cell migration in SK-Hep1 and SNU-387 cells.

### PGCP knockdown induces LRP6 phosphorylation and activates Wnt/β-catenin signaling

**Figure 3:**
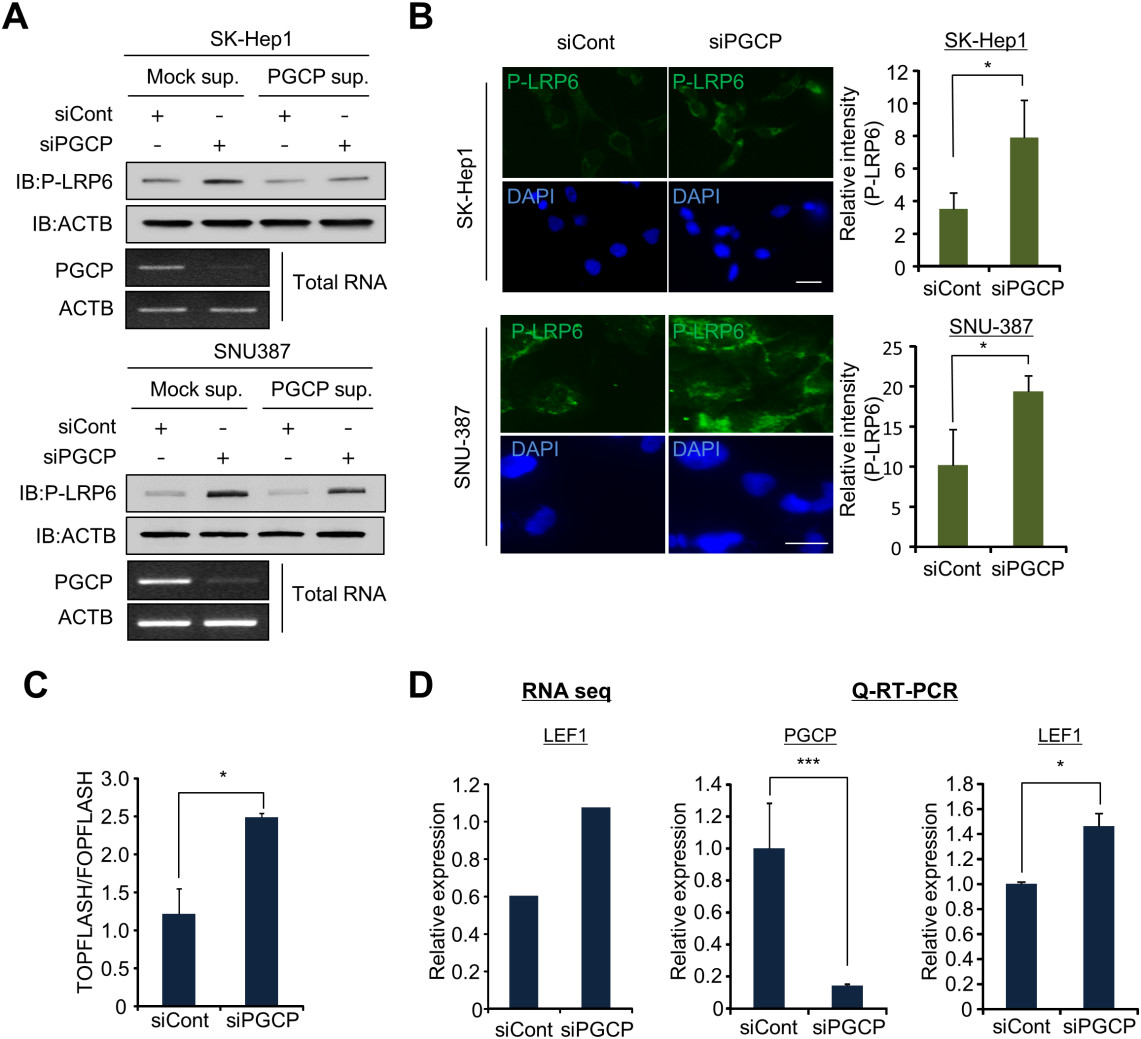
PGCP knockdown induces phosphorylation of LRP6 **A.** Detection of phospho-LRP6 by Western blotting. SK-Hep1 and SNU-387 cells treated with siCont or siPGCP were incubated with supernatant including overexpressed PGCP recombinant proteins for 24 h. Western blot analysis was performed with antibodies against LRP6 phosphorylation. ACTB was used as an internal control. **B.** Immunofluorescence analysis of phospho-LRP6 after treatment with siPGCP and siCont. DAPI was used to counterstain nuclei. The signal intensity corresponding to phospho-LRP6 was quantified using the ImageJ software (Right). The p values were calculated using Student's t-test (*p<0.05, ***p<0.001). **C.** TOPFLASH and FOPFLASH analysis in SK-Hep1 after treatment with siCont or siPGCP. The results are expressed as the ratio of TOPFLASH over FOPFLASH activity, and the p value was calculated using Student's t-test (*p<0.05). **D.** Relative expression of LEF1 analyzed by RNA-seq (left), and validation of RNA-seq data using quantitative RT-PCR analysis (right). The p values were calculated using Student's t-test (*p<0.05, ***p<0.001).

To identify upstream signals of GSK3β regulated by PGCP, we used a candidate analysis approach. Various upstream proteins, such as ERK, mTOR and Wnt, are involved in the inactivation of GSK3β and tumor metastasis [24]. In the Western blot analysis, we observed a significant increase in the amount of phospho-LRP6 after treatment with siPGCP, but phosphorylation of ERK and mTOR did not change between the cells treated with siCont or siPGCP (Supplementary Figure S5). As LRP6 is a co-receptor for Wnt in the canonical Wnt/β-catenin signaling, and the up-regulation of LRP6 phosphorylation induces tumor metastasis [10, 11], we next examined the regulation of phospho-LRP6 by PGCP. The addition of the PGCP supernatant suppressed the LRP6 phosphorylation in both SK-Hep1 and SNU-387 cells (Figure 3A). Additionally, in the immunocytochemistry analysis, the phosphorylation of LRP6 was significantly induced in the PGCP knockdown cells (Figure 3B). Furthermore, in TOPFLASH/FOPFLASH analysis for the evaluation of β-catenin-dependent signaling, the cells treated with siPGCP showed significantly enhanced TOPFLASH reporter activity (Figure 3C), indicating that PGCP negatively regulates Tcf-4/β-catenin activity. It has been reported that the Wnt/β-catenin target gene LEF1 mediates lung adenocarcinoma metastasis [11]. Therefore, we examined LEF1 expression levels using RNA-seq. We observed an increase in LEF1 expression as a result of PGCP knockdown. This result was validated by quantitative RT-PCR analysis after treatment with siPGCP and siCont (Figure 3D). In addition, to investigate the expression levels of β-catenin-related genes by PGCP knockdown in more detail, we collected a list of β-catenin-related genes and re-analyzed their RNA-seq data in PGCP knocked down SK-Hep1 cells. Among 175 genes, 54% of the genes were upregulated by the knockdown of PGCP, only 29% of the genes were down-regulated (Supplementary Figure S6). Taken together, these results suggest that PGCP knockdown up-regulates Wnt/β-catenin signaling through the induction of LRP6 phosphorylation, leading to increased liver cancer metastasis.

### The expression of DKK4 for blocking Wnt/β-catenin signaling is regulated by PGCP

**Figure 4:**
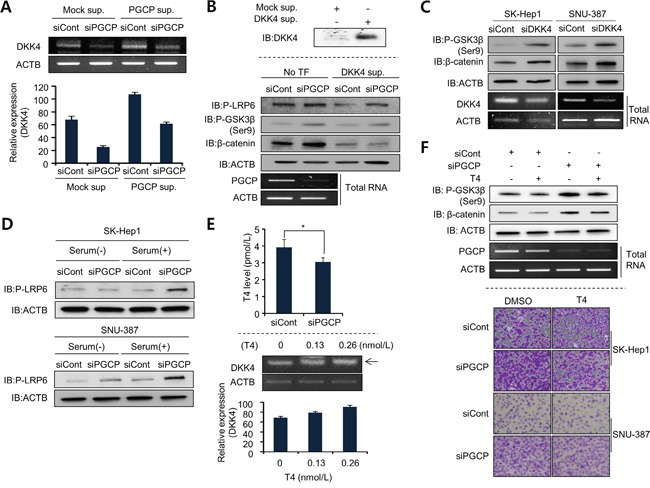
PGCP transcriptionally modulates DKK4 expression in a T4-dependent manner **A.** Semi-quantitative RT-PCR analysis of DKK4 expression in SK-Hep1 cells treated with PGCP supernatant after PGCP knockdown. ACTB was used as an internal control (top). The signal intensity corresponding to DKK4 was quantified using the ImageJ sofware (bottom). **B.** Western blot analysis after the DKK4 addition to PGCP knockdown cells. Top, Western blot analysis after transfection with FALG-Mock or FLAG-DKK4, respectively. After 24 h, the culture media including overexpressed DKK4 recombinant proteins was concentrated using an Amicon column. The samples were immunoblotted with anti-DKK4 antibody. Bottom, SK-Hep1 cells were treated with siRNAs (siCont and siPGCP) for 48 h, and secreted DKK4 was added to the culture media. After 24 h, Western blot analysis was performed using the indicated antibodies. ACTB was used as an internal control. **C.** Detection of phospho-GSK3β (Ser9) and β-catenin after siDKK4 silencing in SK-Hep1 and SNU-387 cells. Western blot analysis was performed using the indicated antibodies. ACTB was used as an internal control. **D.** Detection of phospho-LRP6 by Western blotting. SK-Hep1 and SNU-387 cells treated with siCont or siPGCP were grown with or without serum. Whole cell lysates were analyzed by Western blotting using the indicated antibodies. ACTB was used as a loading control. **E.** T4 assay using a Human Free Thyroxine ELISA kit. SK-Hep1 cells treated with siCont or siPGCP were cultured for 48 h. The p value was calculated using Student's t-test (*p<0.05) (top). Quantitative RT-PCR analysis of DKK4 mRNA level after T4 treatment for 24 h. ACTB was used as a loading control. The signal intensity corresponding to DKK4 was quantified using the ImageJ software (bottom). **F.** Detection of phospho-GSK3β (Ser9) and β-catenin. Western blot analysis was performed after treatment with T4 (final 100 ng/ml) in siRNA-treated SK-Hep1 cells using the indicated antibodies. ACTB was used as an internal control (top). Cell migration assay after T4 treatment. Cells treated with siCont or siPGCP were incubated with T4 (final 100 ng/ml) for 12 h before cell migration assay. DMSO was used as a negative control (bottom).

DKK4 is positively regulated by T3 and TR in liver cancer [14, 15], and PGCP liberates T4 from the N-terminus of Tg [19]. In the cell, T4 is converted to the active T3 by deiodinases [25]. Thus we considered the possibility that T4 liberated by PGCP may increase DKK4 expression and block Wnt/β-catenin signaling. To confirm our hypothesis, we performed semi-quantitative RT-PCR analysis of DKK1 and DKK4 mRNA levels in SK-Hep1 and SNU-387 cells treated with siPGCP. The results showed that PGCP knockdown reduced DKK4 but not DKK1 expression (Supplementary Figure S7A and S7B). The addition of PGCP supernatant to the cells treated with siPGCP restored the expression level of DKK4 (Figure 4A). These results indicate that PGCP upregulates DKK4 expression in SK-Hep1 and SNU-387 cells.

Because DKK4 is also a secretory protein, we added the supernatant from 293T cells overexpressing DKK4 to the SK-Hep1 cells treated with siCont or siPGCP, and then compared the levels of p-LRP6, p-GSK3β and β-catenin. The presence of DKK4 protein in culture medium abolished the PGCP knockdown-mediated induction of phospho-LRP6 and cell migrations, inactivation of GSK3β, and increase in β-catenin levels causing inactivation of Wnt/β-catenin signaling (Figure 4B and Supplementary Figure S7C). GSK3β phosphorylation and β-catenin stability were also increased after siDKK4 treatment as shown by the depletion of PGCP (Figure 4C). Furthermore, DKK4 knockdown enhanced cell migration (Supplementary Figure S7D). These findings suggest that the down-regulation of DKK4 expression by PGCP knockdown can affect the Wnt/β-catenin signaling whereby positively contributing to cell metastasis. This is in agreement with the recent reports on the relationship between DKK4 and tumor metastasis [14, 15, 26, 27].

### Thyroxine (T4) negatively regulates PGCP-mediated cell migration and invasion

To analyze whether the effects of PGCP knockdown on cell migration and invasion were dependent on the concentration of T4, and to exclude the contribution of free Tg present in serum, the siPGCP- and siCont-treated SK-Hep1 and SNU-387 cells were cultured with and without serum. In the absence of serum, the phosphorylation of LRP6 was either unaffected (SK-Hep1) or slightly increased (SNU-387) by PGCP knockdown. In contrast, serum supplementation caused a significant induction of LRP6 phosphorylation in both PGCP-depleted tumor cell lines (Figure 4D). Because the regulation of DKK4 is controlled by T4 levels, we also measured the concentration of T4 in the culture media of siCont- or siPGCP-treated cell. In siPGCP-treated cells, T4 concentration was decreased (Figure 4E, top). To confirm the relationships between DKK4 and PGCP in more detail, the SK-Hep1cells were treated with T4. The expression of DKK4 was increased in a T4 dose-dependent manner (Figure 4E, bottom), whereas siPGCP-mediated induction of phospho-GSK3β and β-catenin was decreased by the T4 treatment (Figure 4F, top). Additionally, cell migration analysis revealed that the augmentation of cell migration by PGCP knockdown was significantly reduced by T4 treatment in SK-Hep1 and SNU-387 cells (Figure 4F, bottom). These results suggest that T4 induced by PGCP suppresses the Wnt/β-catenin signaling by down-regulating DKK4 expression.

### Knockdown of PGCP promotes metastasis *in vivo*

**Figure 5:**
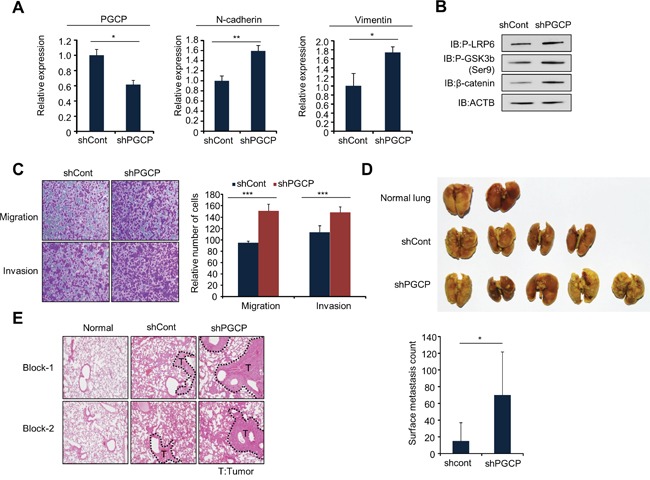
Knockdown of PGCP promotes lung metastasis *in vivo* **A.** Quantitative RT-PCR analysis of PGCP, N-cadherin and vimentin cDNA was isolated from shCont and shPGCP stable cell lines. The p values were calculated using Student's t-test (*p<0.05, **p<0.01). **B.** Western blot analysis of phospho-LRP6, phospho-GSK3β (Ser9) and β-catenin. ACTB was used as an internal control. **C.** Migration and invasion assay with shCont and shPGCP cells. Migrating and invasive cells were fixed with methanol after 24 h stained with crystal violet. The p values were calculated using Student's t-test ***p <0.001. **D.** Gross lung metastases. shCont and shPGCP cells (2 × 10^6^) were injected into BALB/c nude mice through tail vein, and lung metastases were assessed 4 weeks after injection (n=5 per group) (top). Quantification of metastatic nodules (bottom). p values were calculated using Student's t-test *p <0.05. **E.** Representative images of hematoxylin and eosin staining of lung metastases. T, tumor.

To confirm the increase in cell metastasis by PGCP knockdown *in vivo*, we established stable shCont and shPGCP SK-Hep1 cell lines. As expected, PGCP knockdown caused induction of mesenchymal markers (N-cadherin, vimentin) (Figure 5A), up-regulation of phospho-LRP6, phospho-GSK3β and β-catenin in (Figure 5B) and increased cell migration and invasion (Figure 5C). In experimental metastasis assay, one month after intravenous (i.v.) injection of the shCont and shPGCP stable cell lines, the development of the lung metastasis was examined. The shPGCP-SK-Hep1 cells produced more frequent and larger lung metastases upon intravenous injection in immunocompromised mice as compared to the shCont cells (Figure 5D). Also, in hematoxylin and eosin (H&E) stain, the lungs showed that the shPGCP group had significantly more massive solid histological features (Figure 5E). These results suggested that PGCP reduction in human SK-Hep1 cells decreased catalysis of mouse-origin Tg and then production of T4, T3, and DKK4, which increased cell migration and invasion. Together, these data demonstrate that the reduction of PGCP expression derivatives metastasis effects on cancer cells.

## DISCUSSION

Canonical Wnt/β-catenin signaling is strongly associated with tumor growth and metastasis. When elevated, β-catenin binds to TCF/LEF transcription factors and regulates a number of genes involved in tumorigenic and metastatic processes [9, 10, 28]. Thus, targeting Wnt/β-catenin signaling pathway is a potential avenue for drug development. Currently, celecoxib, a COX2 inhibitor, has been used clinically to inhibit β-catenin-dependent transcription [29]. Additionally, Wnt-blocking antibodies obstructed proliferation and led to cell apoptosis [30]. Therefore, the identification of novel Wnt regulator may be useful for the development of a new targeted therapy.

Here, we provide evidence that PGCP associates with Wnt/β-catenin signaling which mediates metastasis. The evidence was supported by the finding of a reverse correlation between the PGCP and β-catenin expression in human liver cancer tissues. In addition, the down-regulation of PGCP not only induced cell migration/invasion in SK-hep1 and SNU-387 cells but also promoted lung metastasis in a mouse model. Thus, we established that the PGCP function is strongly associated with tumor metastasis in liver cancer. As PGCP is a secretory protein [20, 21], we focused on the relationship between the level of PGCP expression and metastasis. As shown in Figure 1B and Supplementary Figure S2, high concentrations of PGCP protein in the tumor environment inhibited cell migration and invasion. Accordingly, low concentrations of PGCP caused by siRNA treatment initiated tumor metastasis in SK-hep1 and SNU-387 cells. In addition, immunohistochemical data in human cancer tissues revealed the overexpression of PGCP in HCC. In analyzing the data from the Oncomine public database, we also found that the expression of PGCP was significantly increased compared with normal tissue (Supplementary Figure S8A and B left). Moreover, in comparing the PGCP expression levels by tumor grade, the expression level of PGCP gradually decreased according to tumor grade, indicating that the reduction of PGCP expression may promote tumor metastasis (Supplementary Figure S8B right). These data suggest that PGCP plays a role as a metastatic regulator. However, further study is warranted to understand the following: 1) how PGCP expression is regulated; 2) what is the critical concentration of PGCP required for initiation of metastasis; and 3) confirmation of the intrahepatic metastasis by PGCP in our model.

The expression level of LRP6 is up-regulated in liver cancer [31]. LRP6 is cell-surface receptors, directly involving to the Wnt/β-catenin signaling [32]. A constitutively active form of LRP6 promoted cell migration and invasion in liver cancer cell lines, and enhanced tumor formation *in vivo* via transmembrane transduction of Wnt signals. In addition, in LRP6-mice model, LRP6 promoted Wnt signaling and accelerated tumorigenesis in breast cancer [31, 33]. Our results reveal that LRP6 phosphorylation is regulated depending on the PGCP expression levels, indicating PGCP may be a critical molecule to modulate the Wnt/β-catenin signaling via LRP6 phosphorylation. These evidences suggest that PGCP can be a good material for study of liver cancer treatments, because of important roles of LRP6 in liver cancer.

Hypothyroidism is a common disorder of the endocrine system resulting from a deficiency of thyroid hormone. It has been reported in cancer patients as a possible risk factor for liver cancer [34]. Thyroid hormone affected liver cancer progression in experimental animals, and T3 treatment promoted a rapid regression of carcinogenesis and diminished liver cancer metastasis in a rat model [35]. Moreover, the rate of tumor growth was slower in hypothyroid mice, but the tumors became more invasive and significantly more metastatic [36]. However, the molecular mechanisms and metastasis-inducing factors of liver cancer metastasis in the presence of hypothyroidism are not fully understood. In the present study, T4 treatment significantly abolished the up-regulation of cell migration and the activation of Wnt/β-catenin signaling caused by PGCP knockdown (Figure 4F). Therefore, we suggest that PGCP-mediated T4 accumulation inactivate the Wnt signaling pathway via the induction of DKK4 expression. This mechanism could explain how low concentration of Thyroid hormone may promote liver cancer metastasis.

The DKK family is strongly associated with tumor metastasis. DKK4 suppresses cell invasion, whereas DKK1 promotes invasion and metastasis in serous ovarian cancer and liver cancer [15–17]. Many reports indicate that DKK4 expression is very low in HCC [14, 15, 27]. However, in this study, we found an increase in cell migration as a result of the DKK4 knockdown which was reversed by the addition of DKK4 protein to culture medium (Supplementary Figure S7C and S7D). Since DKK4 affects cell migration, the level of DKK4 expression may serve as a metastatic switch critical for tumor metastasis.

**Figure 6:**
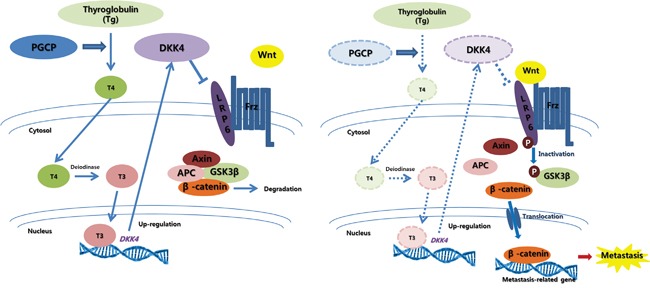
Graphic summary of PGCP effects on liver cancer metastasis Secreted PGCP triggers liberation of T4 from circulating Tg. T4 is converted to T3 by deiodinase, and T3 promotes DKK4 transcription. DKK4, a Wnt antagonist, is also secreted, and prevents the interaction of LRP6 with Wnt-Frizzled complexes by binding to LRP6. This blocks the canonical Wnt signaling, causing β-catenin degradation via Axin-APC-GSK3β complex. In the absence of PGCP function, the levels of T4, T3 and DKK4 are decreased. This activates the canonical Wnt signaling leading to β-catenin nuclear translocation and induction of metastasis-related target genes thereby promoting metastasis.

In conclusion, we report for the first time that PGCP blocks liver cancer metastasis through the inactivation of Wnt/β-catenin signaling by regulating DKK4 expression and thus may represent an effective target for control of liver metastasis (Figure 6). We also suggest that serum concentrations of PGCP or T4 may be used as potential diagnostic markers for the prediction of liver cancer development and metastasis.

## MATERIALS AND METHODS

### Cell culture

The human liver cancer cell line SNU-387 and SK-Hep1 were purchased from the Korean Cell Line Bank (Seoul, South Korea) and was cultured in RPMI and MEM supplemented with 10% fetal bovine serum (FBS) and 1% penicillin/streptomycin in a humidified atmosphere of 5% CO_2_ at 37°C.

### Stable cell lines and siRNA transfection

A lentiviral pool encoding shPGCP (TRCN0000073949) and shRNA control (SHC005) was purchased from Sigma-Aldrich (St. Louis, MO, USA). Viral transduction was performed according to the manufacturer's instructions. The sense sequence of PGCP shRNA is 5′-CCGGCCAGTCTAC TTGATGACTTATCTCGAGA TAAGTCATCAAGTAGACTGGT TTTTG-3′. siRNA duplexes to PGCP, DKK4, and β-catenin were purchased from ST Pharm (Seoul, South Korea), Genosys (Seoul, South Korea) and Genolution (Seoul, South Korea), respectively. siCont (negative control siRNA) was used for control treatment. siRNA sequences are described in the Supplementary Table S1. 100 nM siRNAs were transfected to cancer cell lines using RNAiMAX (Invitrogen, Carlsbad, CA) for 48 h.

### Antibodies and reagents

The anti-CPQ (HPA023235) antibody was purchased from Sigma-Aldrich; anti-N-cadherin (#4061), vimentin (#5741), phospho-GSK-3β (Ser9) (#9323), non-phospho (active) β-catenin (#8814) and phospho-LRP6 (#2568) antibodies were purchased from Cell Signaling Technology (Danvers, MA); anti-β-actin (sc-47778), β-catenin (sc-7963), and HRP-conjugated secondary (sc-2031, sc-2004) antibodies were obtained from Santa Cruz Biotechnology (Santa Cruz, CA). L-thyroxine (T1775) was obtained from Sigma-Aldrich.

### *In vivo* metastasis assay

Cells were harvested at approximately 70% confluence. Female BALB/c nude mice (4 weeks of age) were injected with SK-Hep1 cells (2 × 10^6^/mouse). Cells were injected into the tail veins of mice (n = 5). Animals were sacrificed after 4 weeks, the lungs were fixed in Bouin's solution, and the metastatic nodules were counted.

### Migration and invasion assays

Transwell inserts were coated with a 2% gelatin solution and incubated at RT for 4 h for the migration assay. The gelatin-coated transwell inserts (353097, Falcon BD, Bedford, MA) and invasion chambers (354480, Corning, NY) were rehydrated in serum-free medium. Complete medium with 20% FBS (700 μl) served as a chemoattractant in the bottom chamber, and 5 × 10^4^ cells/well were incubated for 24 h at 37°C with 5% CO_2_. At the end of the incubation period, migrated and invasive cells were fixed with methanol for 5 min and stained with 0.1% crystal violet.

### Western blot analysis

Western blotting was performed according to the manufacturer's instructions [37–39]. The cells were washed once with PBS and then lysed in cold lysis buffer (50 mM Tris-HCl, pH 7.4, 150 mM NaCl, 1% Triton X-100, 0.1% SDS, 1 mM EDTA, 1 mM Na_3_VO_4_, 1 mM NaF, and 1 × protease inhibitor cocktail). Cell lysates were centrifuged at 14,000 × g for 15 min at 4°C and then boiled in 5 × sample buffer. The protein samples were subjected to Western blotting with the indicated antibodies at a 1:500 dilution ratio.

### Immunofluorescence

Immunofluorescence was performed according to the manufacturer's instructions [40]. Cells grown in a 4-well chamber slide (Nalge Nunc, Rochester, NY) were washed two times with PBS and fixed with 4% paraformaldehyde for 30 min at RT. They were permeabilized with 0.5% Triton X-100 in PBS for 5 min at RT. The cells were covered with PBS containing 3% bovine serum albumin for 1 h at RT to block nonspecific hybridization and were then incubated with rabbit anti-vimentin, anti-N-cadherin, anti-phospho-GSK3β (Ser9), anti-β-catenin or anti-phospho-LRP6 at a 1:500 dilution ratio. After being washed with PBS, the cells were stained with an Alexa Fluor 488-conjugated anti-rabbit secondary antibody (A11008, Life Technologies, Carlsbad, CA), an Alexa Fluor 568-conjugated anti-rabbit secondary antibody (A11011, Life Technologies) or an Alexa Fluor 568-conjugated anti-mouse secondary antibody (A11004, Life Technologies) at a 1:100 dilution ratio. Nuclei were counterstained with 4',6'-diamidino-2-phenylindole dihydrochloride (DAPI) (H-1200, Vector Laboratories, Burlingame, CA).

### Semi-quantitative reverse transcription PCR and quantitative RT PCR

Total RNA was isolated from the indicated cell lines using a Qiagen RNeasy Mini Kit according to the manufacturer's instructions [41, 42]. RNA aliquots of 1 μg were then reverse transcribed using the iScript™ cDNA synthesis kit (Bio-Rad, Hercules, CA), according to the standard protocols. For semi-quantitative RT-PCR, cDNA was used as a template for PCR using AccuPower® ProFi Taq PCR PreMix (Bioneer, Daejeon, South Korea). For quantitative RT-PCR, PCR reactions were performed using the CFX96 Real-Time System (Bio-Rad) following the manufacturer's instructions. When it is difficult to detect PGCP expression at endogenous levels by Western blot analysis, real-time PCR analysis was performed. All of the primer sequences are described in Supplementary Table S1.

### Free thyroxine (T4) assay

The T4 assay was performed according to the manufacturer's instructions. The concentration of T4 was measured by the Human Free Thyroxine (FT4) ELISA kit (CSB-E05078h, CUSABIO, Wuhan, China). After treatment of SK-Hep1 cell lines with siCont or siPGCP for 48 h, the supernatant was collected, and the thyroxine assay was performed.

## SUPPLEMENTARY DATA



## Abbreviations

PGCP: plasma glutamate carboxypeptidase
EMT: epithelial mesenchymal transition
MET: mesenchymal epithelial transition
siCont: si-Control
N-cad: N-cadherin
Vim: Vimentin
sup: supernatant
CHX: cycloheximide
β-cat: β-catenin
Q-RT-PCR: quantitative Real-Time PCR
T4: thyroxine
Tg: thyroglobulin
T3: triiodothyronine
