# Supplementary material for: Plasma glutamate carboxypeptidase is a negative regulator in liver cancer metastasis
Source: Oncotarget. 2016 Oct 28;7(48):79774–86. doi: 10.18632/oncotarget.12967 (PMC5340238; doi:10.18632/oncotarget.12967)
Supplement: Supplementary file 1 [file oncotarget-07-79774-s001.pdf]

Plasma glutamate carboxypeptidase is a negative regulator in liver cancer metastasis

SUPPLEMENTARY DATA

Cell viability assay

Cell viability was assessed using a 3-(4,5-dimethylthiazol- 2-yl)-2,5-diphenyl tetrazolium bromide (MTT, Sigma Chemical Co., St. Louis, USA) assay following the manufacturer's instructions. The cells were seeded at a density of 1 × 10^4 cells per well and treated with siCont or siPGCP. MTT (2 mg/ml) was added to each well and the absorbance was measured using a microplate reader (Bio-Rad, Hercules, USA) at 570 nm.

Supernatant preparation and concentration

293T cells were transfected with mock vector, PGCP or DKK4 plasmid in serum-free media. After 48 h, each supernatant was harvested and concentrated using Amicon® Ultra concentrators (UFC903024, Millipore, Darmstadt, Germany). We added up to 14 ml of media to the Amicon® Ultra filter device, and samples were centrifuged at 4,000 × g for 5 min.

Immunohistochemical staining

An EnVision$^+$ kit/HRP kit (Dako, Carpinteria, CA) was used. Paraffin-embedded sections of lung tumor specimens were processed in a microwave (90°C) in an antigen-retrieval solution (pH 9) (S2367; Dako), treated with a peroxidase-blocking reagent, and then treated with a protein-blocking reagent (K130, X0909; Dako). Tissue sections were incubated with rabbit anti-PGCP, anti-β-catenin and anti-phospho-LRP antibodies, followed by incubation with an HRP-conjugated secondary antibody (Dako). Antigens were visualized with the substrate chromogen (Liquid DAB Chromogen; Dako). Finally, tissue specimens were stained with Mayer's hematoxylin solution (Hematoxylin QS; Vector Laboratories) for 20 seconds to discriminate the nucleus from the cytoplasm. Human HCC tissues were purchased from Super Bio Chips (CS5, Seoul, South Korea).

Reporter gene assay

Luciferase activity was examined using a luciferase assay kit (E1910, Promega, Madison, WI). In brief, SK-Hep1 cells were transfected with control siRNA or PGCP siRNA for 48 h and then transfected with TOP- or FOP-Flash reporter constructs and *Renilla* in a 48-well plate (3 × 10^4 cells per well). After 24 h, lysates of SK-Hep1 cells were measured using the assay kit protocol. All of the data were normalized to *Renilla* activity.

RNA-seq experiments and analysis

The quality and integrity of the RNA were confirmed by agarose gel electrophoresis and ethidium bromide staining, followed by visual examination under ultraviolet light. The sequencing library was prepared using the TruSeq RNA Sample Preparation kit v2 (Illumina, San Diego, CA) according to the manufacturer's instructions. In brief, mRNA was purified from total RNA using poly-T oligo-attached magnetic beads, fragmented, and converted into cDNA. Adapters were then ligated to the cDNA, and the fragments were amplified by PCR. Sequencing was performed in paired-end reads (2 x 100 bp) using a Hi-Seq 2000 (Illumina). The reference genome sequence data from *Homo sapiens* were obtained from the University of California Santa Cruz Genome Browser Gateway (assembly ID: hg19). The reference genome index was built using the build component of Bowtie2 (ver. 2.0) and SAMtools (ver. 0.1.18). Tophat2 was used to map reads from the tissue samples to the reference genome (ver. 2.0).

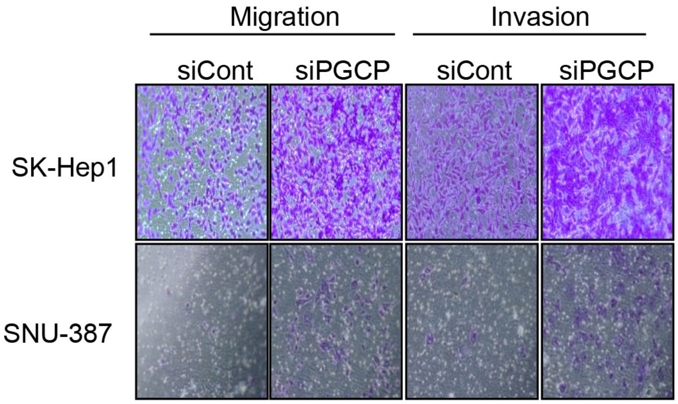

Supplementary Figure S1: PGCP knockdown promotes cell migration and invasion in SK-Hep1 and SNU-387 cells. Cells transfected with the indicated siRNAs were loaded into transwell inserts and invasion chambers, respectively. Migrated and invasive cells were fixed with methanol and stained with crystal violet.

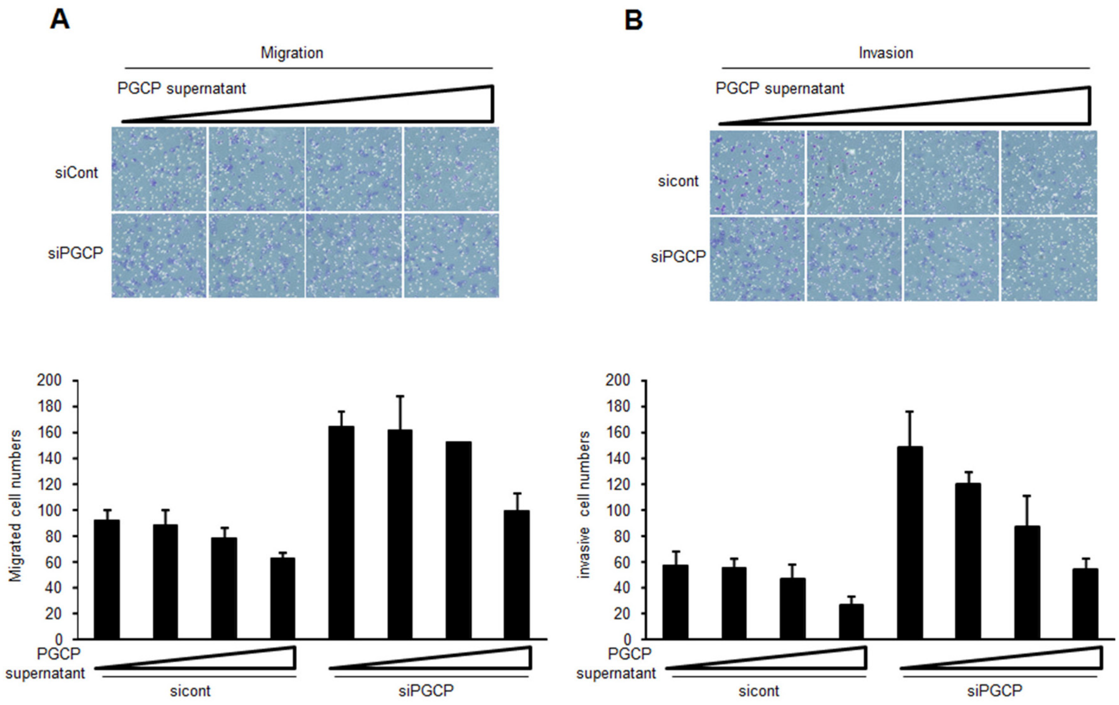

Supplementary Figure S2: The increases in cell migration and invasion ability by siPGCP is abolished by PGCP in a dose-dependent manner. **A** and **B.** Top: SK-Hep1 cells incubated with PGCP supernatant for 24 h after treatment with siRNAs were loaded into transwell inserts and invasion chambers, respectively. Migrated (A) and invasive (B) cells were fixed with methanol and stained with crystal violet. bottom: A graph of migrated and invasive cells.

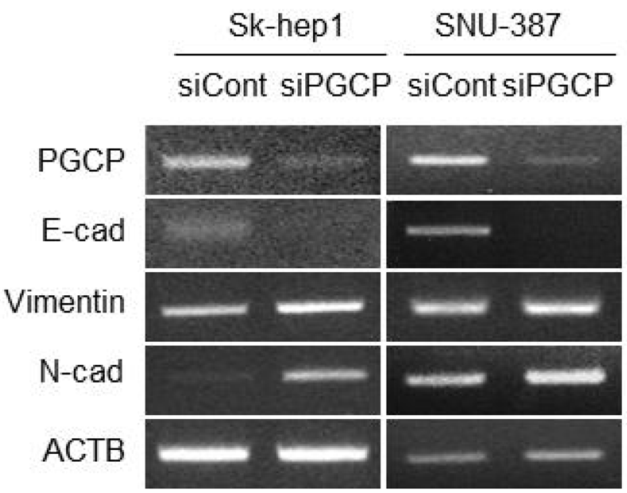

Supplementary Figure S3: Knockdown of PGCP regulates EMT and MET markers. Semi-quantitative RT-PCR for E-cadherin, Vimentin and N-cadherin after treatment with siCont or siPGCP. ACTB was used as an internal control.

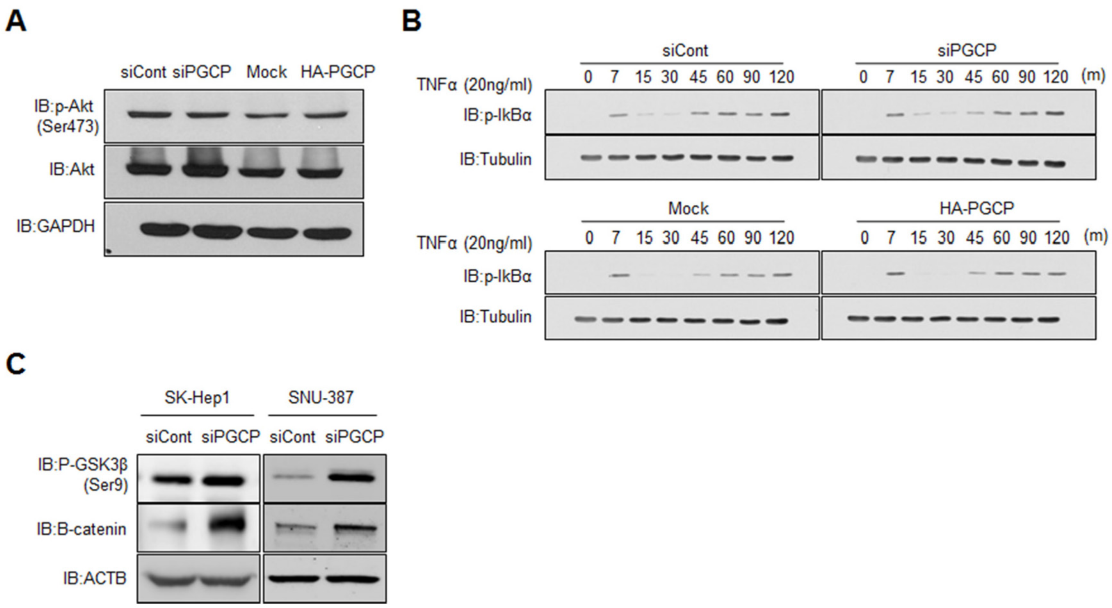

Supplementary Figure S4: PGCP knockdown increases in phosphorylation of GSK3β and stabilize β-catenin. A. Total lysates of the cells treated with siCont, siPGCP, Mock and pHA-PGCP were analyzed by western blotting using the indicated antibodies. GAPDH was used as a loading control. **B.** Western blot analysis after treatment with TNF-α (20 ng/ml) was performed with phospho-IkBα antibody. Tubulin was used as a loading control. **C.** Western blot analysis of the cells treated with siCont or siPGCP was performed using the indicated antibodies. The amount of ACTB was used as an internal control.

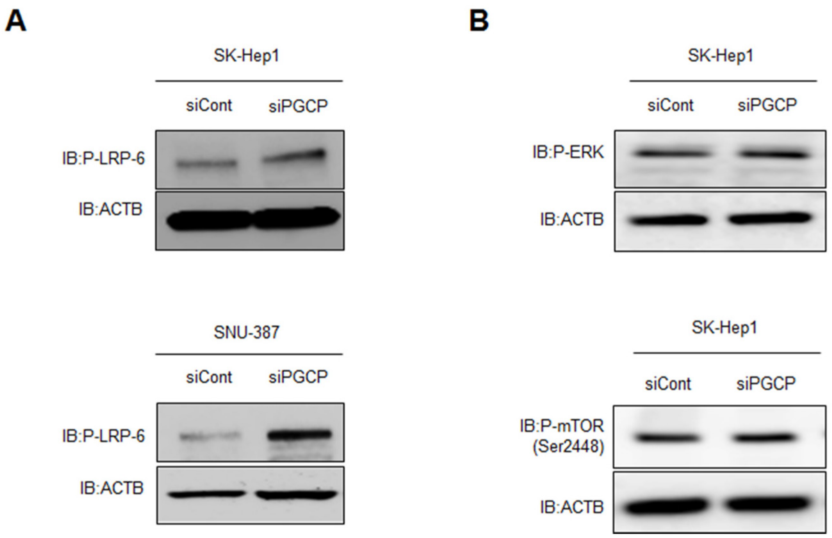

Supplementary Figure S5: LRP6 phosphorylation for Wnt signaling is regulated by PGCP. A. Western blot analysis was performed with antibodies against LRP6 phosphorylation. The amount of ACTB was used as an internal control. **B.** Total lysates of SK-Hep1 cells treated with siCont or siPGCP were analyzed by western blotting using antibodies against the indicated proteins. ACTB was used as a loading control.

A

Gene Symbol	Gene Symbol	Gene Symbol	Gene Symbol
SLC22A4	SLC6A12	CTNNBL1	CDK6
BMP4	CYP8B1	ACTN2	AR
MME	TNFRSF19	DCXR	PTPRG
NUDT6	PREB	SLC1A2	ALDH1L1
BIK	HIBADH	ADH6	INSIG2
FITM2	GPAM	C1orf112	RAB11FIP2
EBPL	SLC22A11	CCDC170	RTP3
HTR2B	HRASLS2	ABCB11	TMEM245
CRLS1	PHLPP1	ACE2	IRS1
TRIB2	TTPA	AQP9	TTC30A
STRA13	MAP3K8	CST1	TENM2
AOX1	LINC00176	CTNNA2	SDPR
BOK	SLC16A1	DSG1	GSTM2
GNAI1	HLF	FAM3B	SLC25A30
MERTK	ESRRG	FIGF	ASAP2
GLUL	NUBPL	FLJ22763	SLC4A4
NEK3	GFRA1	GLYAT	CCDC53
ASPSCR1	ACSS3	HSD11B1	TBX3
CDC14B	FAM169A	KCNJ8	SLCO1B1
VLDLR	SLC2A12	LPPR1	ADRB2
GRHPR	DNAJC12	ODAM	ALDH3A1
ZNRF3	SEC14L2	PAGE4	PDK4
TBCK	CD36	REG1A	C1orf53
CPPED1	UST	REG3A	HHAT
ITPR2	TTC9	RHBG	PRR5L
ANKFN1	RBP1	SALL1	BAMBI
SLC5A6	PANX1	SELENBP1	CAP2
HEPACAM	FAM8A1	SLC17A1	FAS
FRMD3	ACSM3	SMPX	CYP2E1
FAM35A	SPRYD7	SPARCL1	IRX3
MTHFD1	HABP4	SRD5A2	AQP11
LGR5	AMACR	SULT1B1	ECM2
ACSL5	EPHB2	TMEM100	DPP4
PHYHIPL	ADRBK2	UBXN10	SNAI2
HIST1H2AE	CYP1A1	ZNF385B	HOGA1
SLC16A4	TSPAN5	SLC16A10	PDK1
FGF13	DYNC1I1	CORIN	SGK223
THBS4	ABCG2	RUNDC3B	ALDH3A2
EXPH5	ABHD6	NKD1	SLC47A1
SLC16A11	AADAC	MYRIP	TMEM64
TPRG1	NAGS	AQP6	HSDL2
ACSL6	AXIN2	CLDN2	CYP3A
HPD	GPHN	YPEL1	FLJ39653
SLC13A3	RHOBTB1	KCNK1	

B

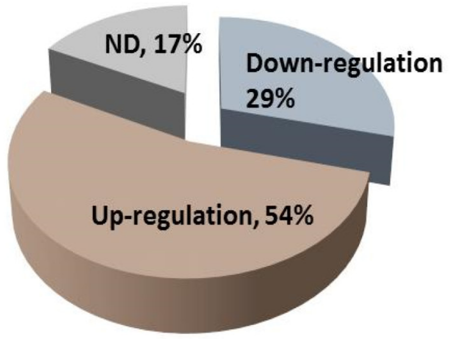

Supplementary Figure S6: Up-regulation of β-catenin related genes by PGCP knockdown in SK-Hep1 cells. A. The gene list of β-catenin related genes in liver cancer (ww.broadinstitute.org/gsea/msigdb/geneset_page.jsp?geneSetName=CHIANG_ LIVER_ CANCER_SUBCLASS_CTNNB1_UP). **B.** Distribution of β-catenin related genes after knockdown of PGCP in SK-Hep1 cells.

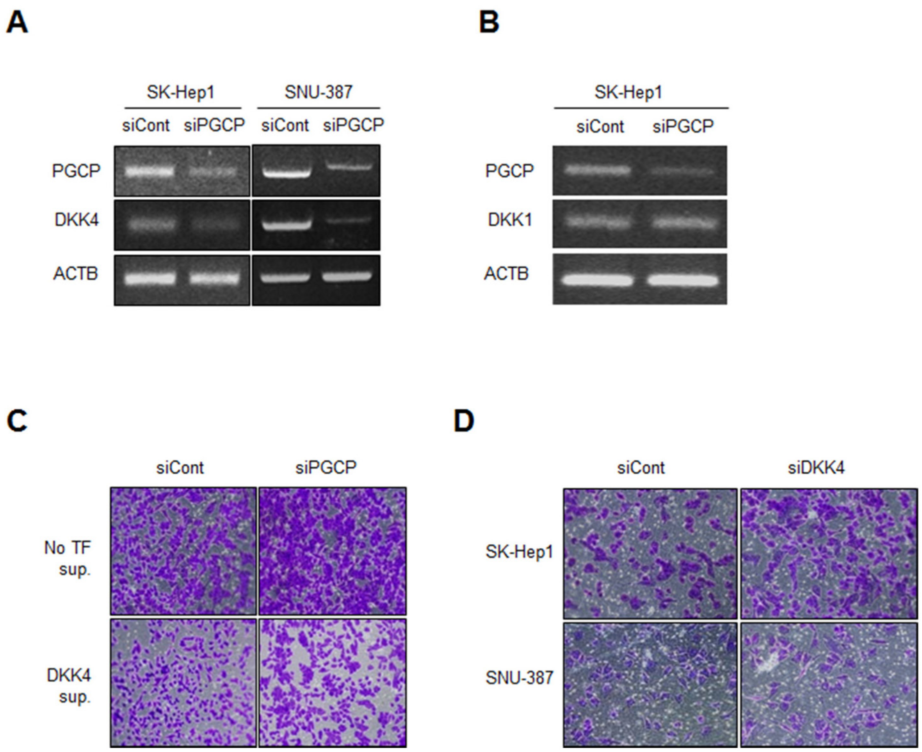

Supplementary Figure S7: DKK4 is regulated by PGCP: A and **B.** Semi-quantitative RT-PCR for detection of DKK4 (A) and DKK1 (B) expression after treatment with siCont or siPGCP. ACTB was used as an internal control. **C** and **D.** Migration assay of cells treated with siPGCP and culture media including overexpressed DKK4 recombinant protein (C) and siDKK4 (D). Migrated cells were fixed with methanol and stained with crystal violet. (No TF; No Transfection).

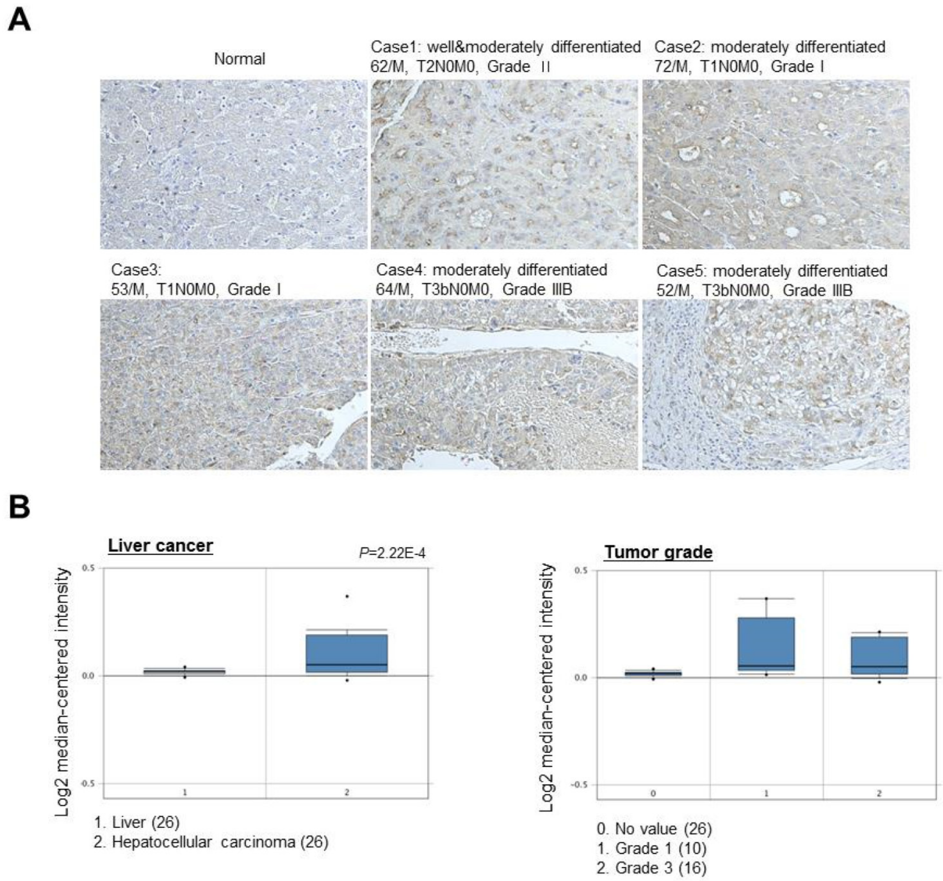

Supplementary Figure S8: Overexpression of PGCP in liver cancers. A. Immunohistochemical analysis of PGCP. Liver cancer samples were purchased from SUPER BIO CHIPS. **B.** Expression of PGCP in liver cancer. Gene expression data in Oncomine (University of Michigan, Ann Arbor, MI) were analyzed.

Supplementary Table S1: Sequences for primers and siRNA

Gene name	Primer sequence
ACTB F	5'-CAAGAGATGGCCACGGCTGCT-3'
ACTB R	5'-TCCTTCTGCATCCTGTCGGCA-3'
DKK1 F	5'-AGCACCTTGGATGGGTATTC-3'
DKK1 R	5'-GTATCCGGCAAGACAGACCT-3'
DKK4 F	5'-CCAGCGAGATGCCATGTG-3'
DKK4 R	5'-TGCATCTTCCATCGTAGTACAAA-3'
LEF1 F	5'-AGAACACCCCGATGACGGA-3'
LEF1 R	5'-GGCATCATTATGTACCCGGAAT-3'
E-cad F	5'-GGTTCAAGCTGCTGACCTTC-3'
E-cad R	5'-AGCCAGTTGGCAGTGTCTCT-3'
N-cad F	5'-ACAGTGGCCACCTACAAAGG-3'
N-cad R	5'-TGATCCCTCAGGAACTGTCC-3'
PGCP F	5'-AGCGATTGGCACTTCTGG-3'
PGCP R	5'-TCGGTATTGCACCGTCCT-3'
Vim F	5'-ATGTCCACCAGGTCCGTG-3'
Vim R	5'-TCGTTGGTTAGCTGGTCCAC-3'
Control siRNA	5'-AUGAACGUGAAUUGCUCAATT-3'
	5'-UUGAGCAAUUCACGUUCAUTT-3'
PGCP siRNA	5'-CAAUCAUCAACCUAGCUGUTT-3'
	5'-ACAGCUAGGUUGAUGAUUGTT-3'
B-catenin siRNA	5'-CUGGGACCUUGCAUAACCUTT-3'
	5'-AGGUUAUGCAAGGUCCCAGTT-3'
DKK4 siRNA	5'-GACACUCUGUGUGAACGAUTT -3'
	5'-AUCGUUCACACAGAGUGUCTT -3'
